# Direct and indirect targeting of MYC to treat acute myeloid leukemia

**DOI:** 10.1007/s00280-015-2766-z

**Published:** 2015-05-09

**Authors:** Sam Brondfield, Sushma Umesh, Alexandra Corella, Johannes Zuber, Amy R. Rappaport, Coline Gaillard, Scott W. Lowe, Andrei Goga, Scott C. Kogan

**Affiliations:** Department of Laboratory Medicine and Helen Diller Family Comprehensive Cancer Center, University of California San Francisco, 513 Parnassus Avenue, Room S-561, Box 0451, San Francisco, CA 94143-0451 USA; Department of Medicine, University of California San Francisco, San Francisco, CA USA; Department of Cell and Tissue Biology and Department of Medicine, University of California San Francisco, San Francisco, CA USA; Research Institute of Molecular Pathology, Vienna, Austria; Memorial Sloan-Kettering Cancer Center and Howard Hughes Medical Institute, New York, NY USA; Rollins School of Public Health, Emory University, Atlanta, Georgia; Department of Molecular and Cellular Biology, University of Washington, Seattle, WA USA; Genentech Inc., South San Francisco, CA USA

**Keywords:** MYC, AML, Myeloid leukemia, JQ1, BRD4

## Abstract

**Purpose:**

Acute myeloid leukemia (AML) is the most common acute leukemia in adults and is often resistant to conventional therapies. The *MYC* oncogene is commonly overexpressed in AML but has remained an elusive target. We aimed to examine the consequences of targeting MYC both directly and indirectly in AML overexpressing *MYC/Myc* due to trisomy 8/15 (human/mouse), *FLT3*-*ITD* mutation, or gene amplification.

**Methods:**

We performed in vivo knockdown of *Myc* (shRNAs) and both in vitro and in vivo experiments using four drugs with indirect anti-MYC activity: VX-680, GDC-0941, artemisinin, and JQ1.

**Results:**

shRNA knockdown of *Myc* in mice prolonged survival, regardless of the mechanism underlying MYC overexpression. VX-680, an aurora kinase inhibitor, demonstrated in vitro efficacy against human MYC-overexpressing AMLs regardless of the mechanism of MYC overexpression, but was weakest against a *MYC*-amplified cell line. GDC-0941, a PI3-kinase inhibitor, demonstrated efficacy against several MYC-overexpressing AMLs, although only in vitro. Artemisinin, an antimalarial, did not demonstrate consistent efficacy against any of the human AMLs tested. JQ1, a bromodomain and extra-terminal bromodomain inhibitor, demonstrated both in vitro and in vivo efficacy against several MYC-overexpressing AMLs. We also confirmed a decrease in MYC levels at growth inhibitory doses for JQ1, and importantly, sensitivity of AML cell lines to JQ1 appeared independent of the mechanism of MYC overexpression.

**Conclusions:**

Our data support growing evidence that JQ1 and related compounds may have clinical efficacy in AML treatment regardless of the genetic abnormalities underlying MYC deregulation.

**Electronic supplementary material:**

The online version of this article (doi:10.1007/s00280-015-2766-z) contains supplementary material, which is available to authorized users.

## Introduction

Acute myeloid leukemia (AML) accounts for 80 % of adult acute leukemias [1], and the primary treatment for patients remains combination chemotherapy. However, the rate of complete response to induction chemotherapy falls below 50 % for elderly patients [2]. Even in younger patients, 40 % do not survive beyond two years following diagnosis [3] and multidrug resistance is seen in 33–57 % of AML cases [4]. The discovery of all-*trans* retinoic acid as an effective therapy for acute promyelocytic leukemia (APL) revolutionized the treatment of this unique AML subtype [5], but new molecularly targeted therapies are needed to improve the prognosis and treatment of AML more generally.

MYC is an attractive target for cancer therapeutics due to its regulation by multiple, converging signaling cascades. MYC is a transcription factor of the helix-loop-helix–leucine zipper family that regulates many cellular processes, including proliferation, cell cycle progression, differentiation, and apoptosis [6, 7]. Following dimerization with MAX, MYC binds to target E-box sequences in the regulatory regions of many target genes [8], and appropriate MYC levels are therefore critical to ensure normal cell fate decisions. Deregulation of MYC may result in uncontrolled cell proliferation, immortalization, growth factor independence, genomic instability, and escape from immune surveillance [6]. In addition, MYC has been shown to inhibit myeloid cell differentiation [9] and is found overexpressed or amplified in many hematologic and solid malignancies (reviewed in [6, 7]). In animal models, transduction of unfractionated murine bone marrow (BM) cells with *Myc* results in AML development [10], and expression of inducible anti-*Myc* short hairpin RNA (shRNA) in leukemic cells leads to their depletion both in vitro and in vivo [11]. Similarly, expression of a human *MYC* transgene causes AML in mice, and inactivation of the same transgene causes sustained tumor regression [12].

Various mechanisms can account for MYC overexpression in AML, including trisomy 8 resulting in single copy gain of *MYC*, *MYC* amplification, and deregulated expression due to an upstream mutation (for example in *FLT3*, a gene encoding a receptor tyrosine kinase); 9 % of AMLs are characterized by trisomy 8, making it the most common chromosomal abnormality in human AML [13]. This gain leads to increased MYC levels in such leukemias [14], and importantly, our laboratory showed that MYC contributes to the pathogenic effect of trisomy 8 [14]. *MYC* amplifications are more occasional, with double minute chromosomes observed in 1 % of cases [15]. Double minute chromosomes in AML most often include *MYC* [15], and this amplification correlates with higher MYC expression and poorer prognosis [16]. Common mutations upstream of MYC in AML include *FLT3* activating mutations, present in 25–30 % of AML patients and associated with a poor prognosis [17]. As *FLT3* is found upstream of several leukemogenic pathways including Ras and PI3-kinase [18], such mutations could be anticipated to stimulate *MYC* mRNA expression or stabilize MYC protein.

Our laboratory isolated transplantable mouse leukemias that arose in the *hMRP8*-*PML/RARA* transgenic line [17]. In this model, the human *MRP8* promoter controls *PML/RARA*, the fusion gene hallmark of APL, driving its expression in maturing myeloid progenitors, neutrophils, and monocytes. Using these transgenic mice, we identified and generated leukemias that model MYC overexpression via three different mechanisms: single copy gain (resulting from trisomy 15), retroviral expression of an activating mutation of *FLT3*, and retroviral overexpression of MYC as a model of gene amplification. Murine trisomy 15, syntenic to trisomy 8 in humans, is the most common recurring abnormality in this model, paralleling the human data [17]. Importantly, retroviral overexpression of MYC suppresses gain of chromosome 15, suggesting that trisomy 15 largely functions to increase MYC levels [17].

**Fig. 1:**
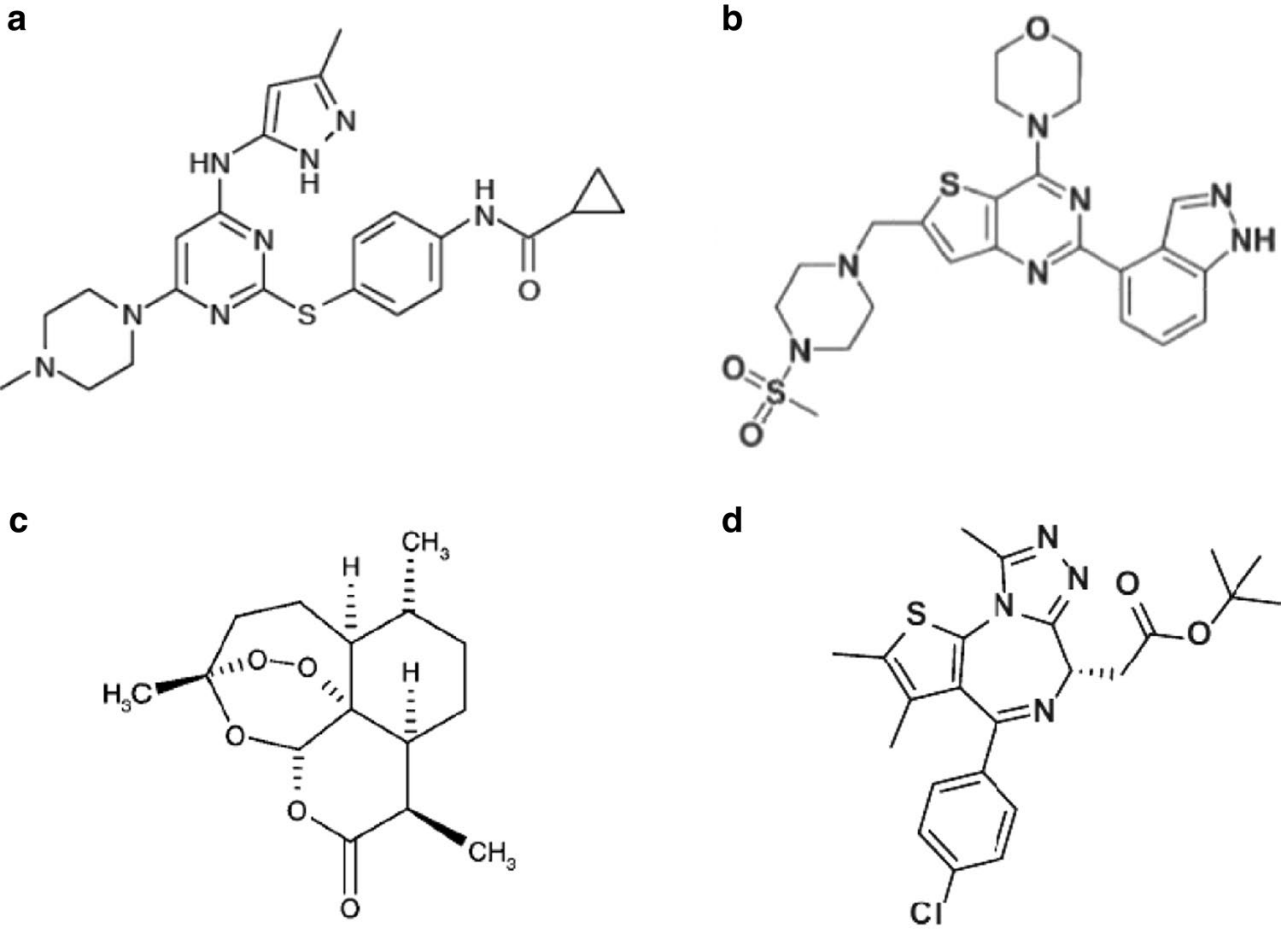
Chemical structures of compounds investigated in our study. **a** VX-680 [19]. **b** GDC-0941 [22]. **c** Artemisinin [24]. **d** JQ1 [26]

Despite its importance in leukemogenesis, MYC has been difficult to target pharmacologically. Most small molecule inhibitors interrupt the MYC:MAX interaction but have shown only mixed results (reviewed in [6]). However, several molecules have demonstrated efficacy in MYC-overexpressing malignancies through indirect targeting of MYC (reviewed in [6, 7]). In this study, we followed a similar strategy to investigate the consequences of targeting MYC indirectly on the growth of AML, using the four compounds described below:

VX-680 (Fig. 1a) belongs to the family of aurora kinase inhibitors [19], which have demonstrated synthetic lethal interactions with overexpressed MYC in prior studies [20, 21]. VX-680 has demonstrated efficacy against AML both in vitro and in vivo [19] and has been recently investigated in a phase I clinical trial for non-AML leukemia patients (Merck, NCT00111683). VX-680 inhibits aurora-A and aurora-B kinases in a p53-independent manner [20], and preliminary data show that it extends survival of mice transplanted with MYC-overexpressing APLs (unpublished data from M. Bishop’s laboratory, University of California San Francisco).

GDC-0941 (Fig. 1b) targets and inhibits PI3-kinase [22], a receptor at the top of a key signaling cascade composed of many downstream effectors, including MYC [23]. When the pathway is activated, the inhibitory phosphorylation of MYC by GSK-3β is released, allowing MYC to be stabilized and translate its effects [23]. Illustrating the broad possible applications of such compound, GDC-0941, is currently undergoing a phase I clinical trial in non-Hodgkin lymphoma and solid cancer patients (Genentech, NCT00876122).

Artemisinin (Fig. 1c) has traditionally been used for its antimalarial properties [24], but recent studies suggest that its use could be extended to tumor treatment [25]. Dihydroartemisinin, the principal active metabolite of artemisinin, has been shown to lead to MYC degradation and induce apoptosis of MYC-overexpressing cells [25] in a GSK-3β-dependent manner.

JQ1 (Fig. 1d) is an inhibitor of the bromodomain and extra-terminal bromodomain (BET) protein BRD4 [26, 27]. Interestingly, JQ1 can also attenuate solid tumor growth without affecting MYC levels, indicating additional MYC-independent effects in some settings [28]. The efficacy of JQ1 has been demonstrated against AML in vitro and in vivo [29], but no clinical trials have been initiated to date.

We hypothesized that the mechanism of MYC overexpression would influence the response to the various MYC-targeting compounds described above, as well as the response to direct MYC-targeting shRNAs. Such information would be useful to stratify patients according to their underlying genetic lesions and personalize AML therapy.

## Materials and methods

### Animals

FVB/n mice were bred and maintained at University of California, San Francisco (UCSF), and their care was in accordance with Institutional Animal Care and Use Committee guidelines.

### Retrovirus production

shRNAs against *MYC/Myc* were previously validated (Lowe laboratory). BOSC23 cells were transfected by gently adding a solution containing CaCl_2_, HBS (pH 7.05), pCL-Eco (helper plasmid), and the shRNA-containing vectors (see “Materials and Methods” in electronic supplementary material for cloning details). After 7 h at 37 °C 8 % CO_2_, the transfection mixture was replaced with fresh media and the plate returned to the incubator. After 48 h, the retrovirus-containing supernatant was harvested, filtered (0.2 μm), and frozen. Three distinct anti-*Myc* and two control (anti-*Renilla* luciferase and anti-*Rpa3*) retroviruses were produced.

### Retroviral transduction

Two independent cryopreserved leukemias were transplanted retro-orbitally into sublethally irradiated (4.5 Gy) recipients: leukemia 1111 (PML/RARA with trisomy 15, designated as “PR/+15”) and leukemia 1127 (PML/RARA with activated *FLT3,* designated as “PR/FLT3_RV_”). Upon euthanasia of sick animals, fresh leukemic bone marrow and spleen cells (in a 1:1 ratio when possible) were harvested, passed through a 70-μm strainer and plated at 1 × 10^6^ cells/well. After spinning, the supernatant was removed, and 1 mL of retrovirus containing 5 % of IL-3 and IL-6 conditioned media and 4 μL of 2 mg/mL polybrene were added/well. The plate was centrifuged at 2500 rpm for 90 min at room temperature and the supernatant replaced with Myelocult M5300 (StemCell Technologies#05300) containing 5 % of IL-3 and IL-6 conditioned media. After 24 h, the transduction procedure was repeated, following which cells were harvested and isolated by flow cytometry.

### Fluorescence activated cell sorting (FACS)

Cells were double-sorted on a BD Biosciences FACSAriaIII. Doublets were eliminated, and DAPI^−^ (Invitrogen cat#D3571, 1:60,000) and mCherry^+^ (and GFP^+^, if applicable) cells were isolated (purity > 95 %); 7000 transduced cells/animal were transplanted back into sublethally irradiated FVB/n CD45.2 recipients following previously described protocols [30, 31].

### Statistics

Survival curves were generated using Prism software (GraphPad) and compared using log-rank analysis. Student’s unpaired *t* test was used to compare the mean percentage of mCherry^+^ cells in BM of euthanized recipients.

### Cell lines origin, culture conditions, and doubling time measurement

BOSC23 cells were maintained in high-glucose Dulbecco’s modified Eagle’s medium (DMEM) containing 10 % FBS; 5637 cells were maintained in RPMI containing 10 % FBS.

MOLM-14, MV4-11, and HL-60 cells were obtained from Dr. Neil Shah’s laboratory at UCSF, which confirmed the presence of* FLT3*-*ITD* in MOLM-14 and MV4-11, and *NRASQ61* in HL-60. These lines were maintained in RPMI containing 10 % FBS. MUTZ-2, OCI-AML5, AP-1060, and FKH-1 cells were authenticated by and ordered from DSMZ (http://www.dsmz.de/). FKH-1 cells were maintained in RPMI containing 20 % FBS. MUTZ-2 cells were maintained in alpha-MEM containing 20 % FBS and 20 % conditioned medium from cell line 5637. OCI-AML5 cells were maintained in alpha-MEM containing 20 % FBS and 10 % conditioned medium from cell line 5637. AP-1060 cells were maintained in Iscove’s MDM containing 20 % FBS and 10 % conditioned medium from cell line 5637. Penicillin–streptomycin and l-glutamine were added to all the cell line media described above. Cell lines were split 2–3 times per week, using split ratios available at http://www.dsmz.de/, and passaged in the Kogan laboratory for less than 6 months. Cell numbers were plotted against time, and a best-fit exponential equation was used to calculate the doubling time for each cell line.

### Drug handling

For in vitro experiments, VX-680 and GDC-0941 (free base) were ordered from LC Laboratories (http://www.lclabs.com/); 99 % of pure artemisinin was ordered from ebiochem (http://www.ebiochem.com/product/artemisinin-99-uv-8157). JQ1 was shipped directly from the laboratory of James Bradner, MD at the Dana-Farber Cancer Institute. Doxorubicin was obtained from the UCSF inpatient pharmacy. For in vivo experiments, GDC-0941 (free base) was obtained directly from the laboratory of Kevin Shannon, MD, and JQ1 was obtained directly from the Bradner laboratory.

### In vitro drug assay and GI50 calculation

To measure the effect of drugs on cell growth, MOLM-14, MV4-11, HL-60, OCI-AML5, and FKH-1, cells were plated in 96-well plates at 10,000 cells/well in 100 μL. For MUTZ-2 and AP-1060, cells were first grown in 24-well plates at 500,000 cells/well in 1 mL before being transferred to 96-well plates in a 1:4 dilution. Drugs were prepared at 60 mM in DMSO (or 600 μM in saline for doxorubicin) and further diluted in half-log serial dilutions. Plates were incubated at 37 °C and 5 % CO_2_, and cell growth measured using the CellTiter-Glo assay (Promega cat#G7570) after two doubling times, per manufacturer’s instructions. Luminescence was measured on a Synergy 2 Multi-Mode Microplate Reader, Biotek model. Relative light units were plotted against drug concentration, and a best-fit logistic curve generated using a four-parameter sigmoidal dose–response model. Data from two independent experiments were averaged to generate the GI50.

To measure the effect of drugs on MYC levels, cells were plated in six well plates at 3 × 10^6^ cells/well and treated as described above for one doubling time. In addition to an untreated control, two drug concentrations were chosen (unless specified in the figure): lower (nearest half-log unit below the calculated GI50) and higher (nearest half-log unit above the calculated GI50).

### Western blots

Following one doubling time of exposure, cells were washed three times with PBS and lysed with protease inhibitors-containing RIPA buffer. After measuring protein concentration using the Bio-Rad DC protein assay, 40 ug of protein was loaded with NuPage LDS sample buffer and NuPage reducing agent, onto a 4–12 % Bis–Tris gradient gel. Protein extracts were transferred to a nitrocellulose membrane, which was blocked with a TBST 5 % milk solution. Anti-c-MYC was added at 1:10,000 (Epitomics cat#1472-1, rabbit monoclonal), followed by goat anti-rabbit IgG-HRP (Santa Cruz cat#SC-2054) at 1:10,000. To detect β-actin, anti-actin (Sigma Aldrich cat#A2228, mouse) was added at 1:10,000, followed by a goat anti-mouse IgG-HRP (Santa Cruz cat#SC-2055) at 1:10,000. Details on imaging and calculations are provided in the "Materials and Methods" in the electronic supplementary material.

### In vivo GDC-0941 experiments

Vehicle solution was prepared with 0.5 % hydroxypropyl methylcellulose in ddH_2_O, to which 500× Tween 80 was added. Each day, a 12.5 mg/mL solution of GDC-0941 was prepared in the above vehicle. In vivo experiments were conducted in collaboration with the UCSF Helen Diller Family Comprehensive Cancer Center Preclinical Therapeutics Core. Groups of five FVB/n mice were sublethally irradiated (4.5 Gy) and retro-orbitally injected with the following four murine leukemias: PR/+15 and PR/FLT3_RV_ (see the “Retroviral transduction” section for description), leukemia#478 (resulting from retroviral *MYC* overexpression, designated as “PR/MYC_RV_”), and leukemia#214 (constitutively stable MYC control, resulting from retroviral T58A mutant *MYC* overexpression, designated as “PR/MYC_RV_^T58A^”). Mice were treated by oral gavage at 10 μL/g of body weight (125 mg/kg) for 21 days beginning on day 14 post-injection. Pharmacokinetics for GDC-0941 at the dose and administration route utilized in our study has been previously described [22]. Control mice were treated with vehicle. Mice were euthanized upon showing signs of morbidity, and necropsy performed to confirm the presence of leukemia.

### In vivo JQ1 experiments

Vehicle solution was prepared with 10 % hydroxypropyl-beta-cyclodextrin. Each day, a 5 mg/mL solution of JQ1 was prepared in the above vehicle. In vivo experiments and euthanasia were performed as described above. Mice were treated by intra-peritoneal injections at 50 mg/kg of animal for 21 days beginning on day 14 post-injection. Pharmacokinetics for JQ1 at the dose and administration route utilized in our study have been previously described [32]. For leukemia 1111 (PR/+15), one animal required euthanasia following a JQ1 intraperitoneal injection and was censored on the date of death. For leukemia 478 (PR/MYC_RV_), three animals in the placebo-treated group and three animals in the JQ1-treated group developed leukemia and were included in the survival analysis (two animals in each group failed to engraft with leukemic cells).

## Results

### Myc knockdown in Myc-overexpressing AMLs prolongs survival in recipient mice and prevents AML cells from predominating in the BM

To determine whether *Myc* knockdown prolongs survival in mice injected with MYC-overexpressing AMLs, leukemic cells isolated from the *hMRP8*-*PML/RARA* model were transduced with an anti-*Myc* shRNA-containing retrovirus [11]. Leukemias 1111 (“PR/+15,” overexpressing MYC through trisomy 15) and 1127 (“PR/FLT3_RV_,” overexpressing MYC via constitutive FLT3 activation) were passaged into recipient animals. Leukemic BM and spleen were harvested fresh from sick animals and transduced with mCherry-tagged *Myc* or control shRNAs [11]. mCherry^+^ cells were double-sorted (mCherry^+^/GFP^+^ cells in the case of the PR/FLT3_RV_ leukemia) and injected into sublethally irradiated 45.2 or FVB/n recipients. Mice were euthanized upon showing signs of morbidity, or 15 weeks post-transplant, and BM was assessed for the presence of mCherry^+^ (and GFP^+^ if applicable) cells.

**Fig. 2:**
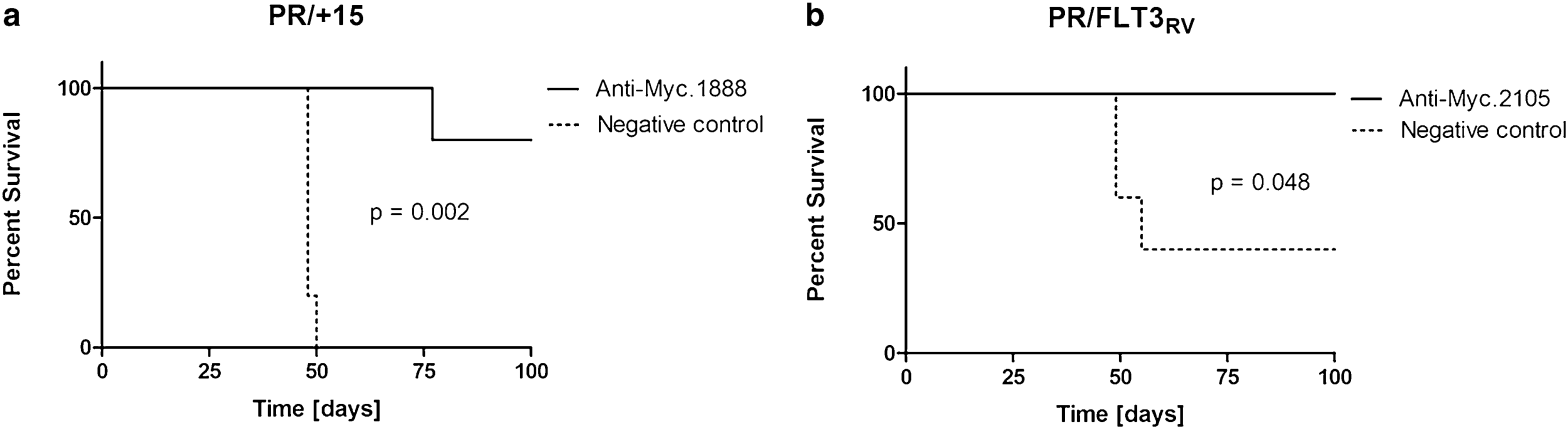
*Myc* knockdown prolongs survival in mice transplanted with *Myc*-overexpressing AML. **a** Kaplan–Meier curve comparing survival of recipient mice injected with leukemia 1111, a PML/RARA leukemia with trisomy 15 (designated as ‘PR/+15’), after shRNA knockdown against *Myc* (or control) and isolation by flow cytometry. *N* = 5 mice per group. **b**, Kaplan–Meier curve comparing survival of recipient mice injected with leukemia 1127, a PML/RARA leukemia obtained by retroviral transduction of PML/RARA BM with activated *FLT3* (designated as ‘PR/FLT3_RV_’), after shRNA knockdown and isolation by flow cytometry. Note that not all animals in the group receiving PR/FLT3_RV_ cells transduced with control vector developed leukemia, likely reflecting the low numbers of cells transplanted. *N* = 5 mice per group

Mice injected with AMLs transduced with anti-*Myc* shRNA survived longer than mice injected with AMLs transduced with anti-*Renilla* luciferase shRNA (Fig. 2a, b), consistent with previous data showing that *Myc* shRNA knockdown could prolong survival in mice injected with murine AML [11]. Furthermore, the effect of MYC knockdown paralleled the survival benefit observed in mice injected with AMLs transduced with anti-*Rpa3* shRNA (data not shown), as *Rpa3* is an essential gene required for DNA replication [33]. BM from mice injected with anti-*Myc*-transduced AML contained very few mCherry^+^ cells at euthanasia, while BM from mice injected with anti-*Renilla*-transduced AML contained a high percentage of mCherry^+^ cells (data not shown), indicating that AMLs transduced with anti-*Myc* shRNA were less able to predominate in vivo. These data suggested that various MYC-overexpressing AMLs may respond to anti-MYC therapy and prompted exploration of MYC inhibition in human AML cell lines.

### VX-680, GDC-0941 and JQ1 inhibit growth of MYC-overexpressing human AMLs in vitro, while artemisinin does not

**Fig. 3:**
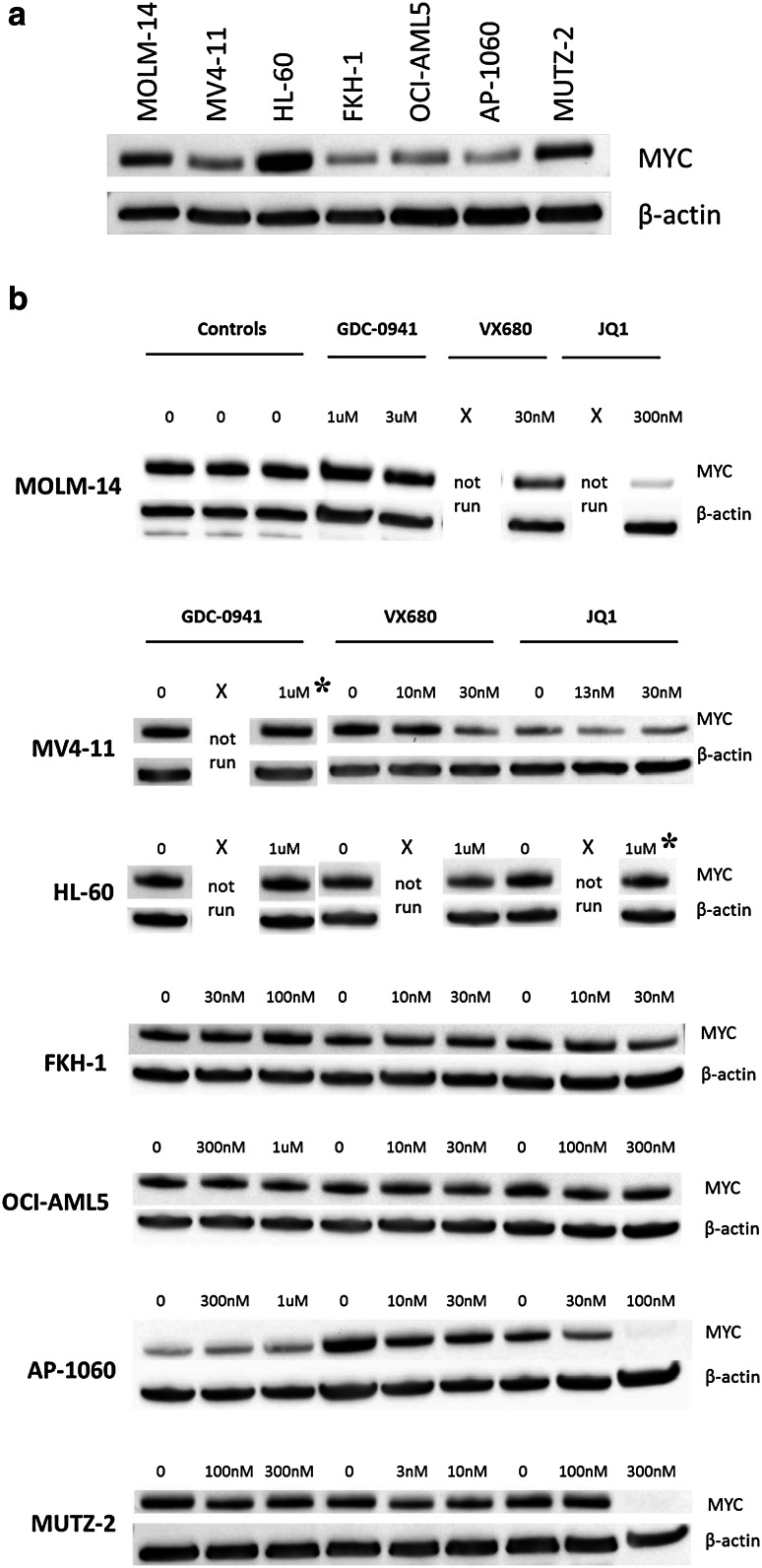
Levels of MYC protein observed in the various cell lines, at basal levels and after exposure to GDC-0941, VX-680, or JQ1. **a** Western blots demonstrating basal MYC levels observed in all cell lines, untreated. Normalized levels to those seen in AP-1060 (lowest expressing cell lines) are shown in Figure S3a in electronic supplementary material. **b** MYC levels following drug treatment at concentrations spanning the GI50 (unless specified, see *asterisk* below). *Note*: in a few instances, only one drug concentration was investigated (above the GI50 in all cases). *Asterisk* in these two cases, the drug concentration tested is not immediately above the GI50 but rather another half-log unit higher, yet no decrease in MYC levels is observed at these higher concentrations (for MV4-11, GI50 for GDC-0941 was 212.7nM; for HL-60, GI50 for JQ-1 was 250.1nM)

To examine the benefit of personalized therapy for human MYC-overexpressing AMLs, it was important to determine whether various human AMLs would respond differently to putative anti-MYC therapies according to their underlying genetic lesion. We obtained five human AML cell lines displaying deregulated *MYC* genes, designated herein as “MYC-overexpressing” (OCI-AML5, MUTZ-2, MOLM-14, MV4-11, and HL-60), as well as two control cell lines not known to overexpress MYC (AP-1060 and FKH-1). Mimicking clinical situations, the five AML cell lines had various genetic abnormalities underlying *MYC* overexpression: single copy gain via trisomy 8 (OCI-AML5 and MUTZ-2), *FLT3*-*ITD* mutation and trisomy 8 (MOLM-14 and MV4-11), or *MYC* amplification (HL-60). Given that anti-*MYC* shRNA is not currently a viable AML treatment and that clinical utility of direct MYC inhibitors has not been demonstrated, we proceeded to test the effect on AML growth of four molecules with indirect anti-MYC activity (VX-680, GDC-0941, artemisinin, and JQ1, described in the "Introduction"). We exposed the cell lines to varying drug concentrations and measured cell growth (Figure S1 in electronic supplementary material) and MYC protein levels (Fig. 3 and Figures S2b–d in electronic supplementary material). Importantly for each cell line, we standardized the length of exposure to two doubling times of unexposed cells for cell growth measurements and to one doubling time for MYC levels by western blot (Supplementary Methods, data not shown). Growth inhibition of 50 % (GI50) was calculated for each drug–AML pairing (Table S1 and Figures S3a–g in electronic supplementary material).

Untreated, the control AP-1060, cell line exhibited the lowest levels of MYC as compared to the other cell lines. The other control cell line FKH-1 showed a nearly twofold increase in MYC levels compared to AP-1060. Further illustrating the variation in baseline MYC levels, OCI-AML5, a cell line with trisomy 8, showed MYC levels slightly higher than AP-1060, but below that of FKH-1. HL-60, a cell line with *MYC* amplification, had the highest MYC levels of all cell lines investigated (Fig. 3a and Figure S2a in electronic supplementary material). Thus, baseline overexpression of MYC in these cell lines did not appear to correlate with mechanisms of overexpression, apart from the multiple copy gain in HL-60 which associated with the highest MYC protein levels of the cell lines tested.

**Fig. 4:**
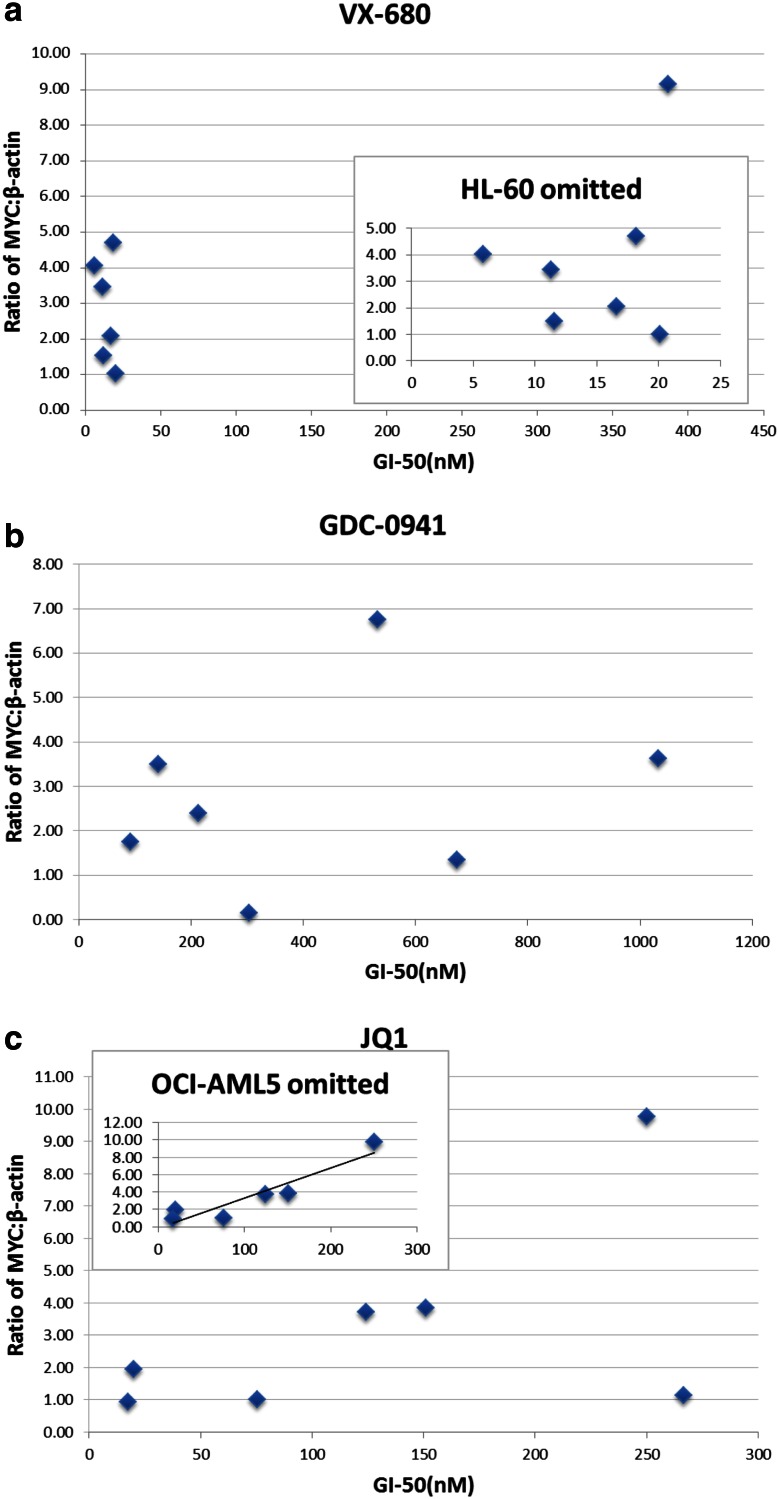
MYC levels of untreated samples were plotted against calculated GI50s for VX-680, GDC-0941, and JQ1 to assess the correlation between MYC expression level and drug sensitivity. **a** HL-60 differed markedly from the other cell lines in regard to sensitivity to VX-680. When HL-60 is omitted, no correlation was observed between GI50 and baseline MYC protein level (*Inset*, *R* ^2^ = 0.12). **b** No correlation was observed between GI50 and baseline MYC protein level when cell lines were treated with GDC-0941 (*R* ^2^ = 0.07). **c** A weak correlation was observed between GI50 and baseline MYC protein level when cell lines were treated with JQ1 (*R* ^2^ = 0.29), trending toward higher GI50 with higher baseline MYC level. OCI-AML5 appeared as an outlier, and a marked correlation emerged when omitting this cell line (*Inset*, *R* ^2^ = 0.86)

The GI50s calculated for each cell line and drug are shown in Tables S1 and S2 (in electronic supplementary material). GI50s for VX-680 were less than or equal to 20 nM for all cell lines except HL-60, which was less than 400 nM, far below the clinically achievable plasma concentration of 5 μM [34]. Of note, there was no correlation observed between drug efficacy and mechanism of MYC overexpression. Given prior work suggesting that VX-680 exhibits synthetic lethality in the presence of MYC overexpression, we did not anticipate a decrease in MYC protein levels near the GI50s, but in fact did see such a decrease for the two FLT3 positive cell lines, MOLM-14 and MV4-11 (Fig. 3b and Figure S2b in electronic supplementary material), which might reflect FLT3 kinase inhibition by VX-680 (see “Discussion”). Notably, although there was no overall correlation between sensitivity to VX-680 and baseline MYC protein levels (Fig. 4a), HL-60, which had the highest MYC protein level of the cell lines tested, appeared to be the most resistant to VX-680.

GI50s for GDC-0941 were less than or equal to 1 μM for all cell lines, also far below the preclinically achievable plasma concentration of 10 μM [22]. A clinical trial assessing oral bioavailability of GDC-0941 in humans has been conducted (NCT01240226), but results are not yet available. Although GDC-0941 demonstrated in vitro efficacy across all cell lines, there was no correlation observed between drug efficacy and mechanism of MYC overexpression (Tables S1 and S2 in electronic supplementary material), and GDC-0941 exposure at levels above the GI50 did not result in a decrease in MYC protein levels in six of the seven cell lines (Fig. 3b and Figure S2c in electronic supplementary material), indicating that the growth inhibitory effects in these six lines are not mediated through decrease in MYC. Further, there was no correlation between sensitivity to GDC-0941 and baseline MYC protein levels (Fig. 4b).

GI50s for artemisinin were less than 2 μM for HL-60 and MV4-11, but above 30 μM for the remaining cell lines, which was the highest drug concentration tested. Importantly, clinically achievable concentrations of artemisinin are approximately of 2 μM (or 530 ng/mL) [35], and given the poor in vitro efficacy observed, western blot data were not generated for artemisinin-treated cell lines.

GI50s for JQ1 were less than 300 nM for all cell lines investigated. Clinically achievable JQ1 concentration has not been verified. As GI50 concentrations were crossed, a decrease in MYC levels was observed, which was >twofold in four of the seven cell lines (Fig. 3b and Figure S2d in electronic supplementary material). Thus, JQ1 demonstrated in vitro efficacy across all cell lines in association with decreasing MYC protein levels. Although there was no correlation observed between drug efficacy and mechanism of MYC overexpression, some correlation was observed between drug efficacy and basal MYC protein level; overall, higher protein level appeared to correlate with decreasing sensitivity to JQ1 (Fig. 4c). OCI-AML5 is an outlier, exhibiting the highest measured GI50 for JQ1 despite having among the lowest measured MYC protein level and showing minimal decrease in MYC level with JQ1 treatment. Removing this datapoint strengthened the correlation between basal MYC protein level and JQ1 resistance (Fig. 4c inset, *R*^2^ = 0.86).

We also performed these experiments using doxorubicin, a cytotoxic chemotherapeutic agent with clinically achievable levels of 100 nM [36]. GI50s for all cell lines were less than or equal to 40 nM (Tables S1 and S2 in electronic supplementary material).

Given our results, the termination of clinical trials using VX-680 due to QTc prolongation and a general lack of encouraging clinical results with that drug [37], we chose to proceed with preclinical in vivo studies using GDC-0941 and JQ1.

### GDC-0941 does not prolong survival in mice transplanted with MYC-overexpressing AMLs

We wished to determine whether the efficacy of GDC-0941 against MYC-overexpressing AMLs we observed in vitro (Table S1 in electronic supplementary material) was also observed in vivo. Groups of five sublethally irradiated mice were transplanted with one of the four murine AMLs: PR/+15, PR/FLT3_RV_, PR/MYC_RV_, and PR/MYC_RV_^T58A^ (see “Introduction” and “Materials and Methods” for descriptions of these leukemias). Starting 14 days after AML injection, mice were treated with GDC-0941 or vehicle for 21 days. Mice treated with GDC-0941 did not survive longer than mice treated with vehicle for any of the four leukemias (data not shown).

### JQ1 prolongs survival in mice transplanted with MYC-overexpressing AMLs

After showing that targeting MYC in murine MYC-overexpressing AMLs prolongs survival (Fig. 2) and that JQ1 demonstrated efficacy against several MYC-overexpressing AML cell lines (Table S1 in electronic supplementary material), we proceeded with preclinical studies using JQ1 in three murine AMLs with mechanisms of MYC overexpression paralleling those seen in human AML. Groups of sublethally irradiated mice were injected with the above AMLs. Starting 14 days after injection, mice were treated with JQ1 or placebo for 21 days.

**Fig. 5:**
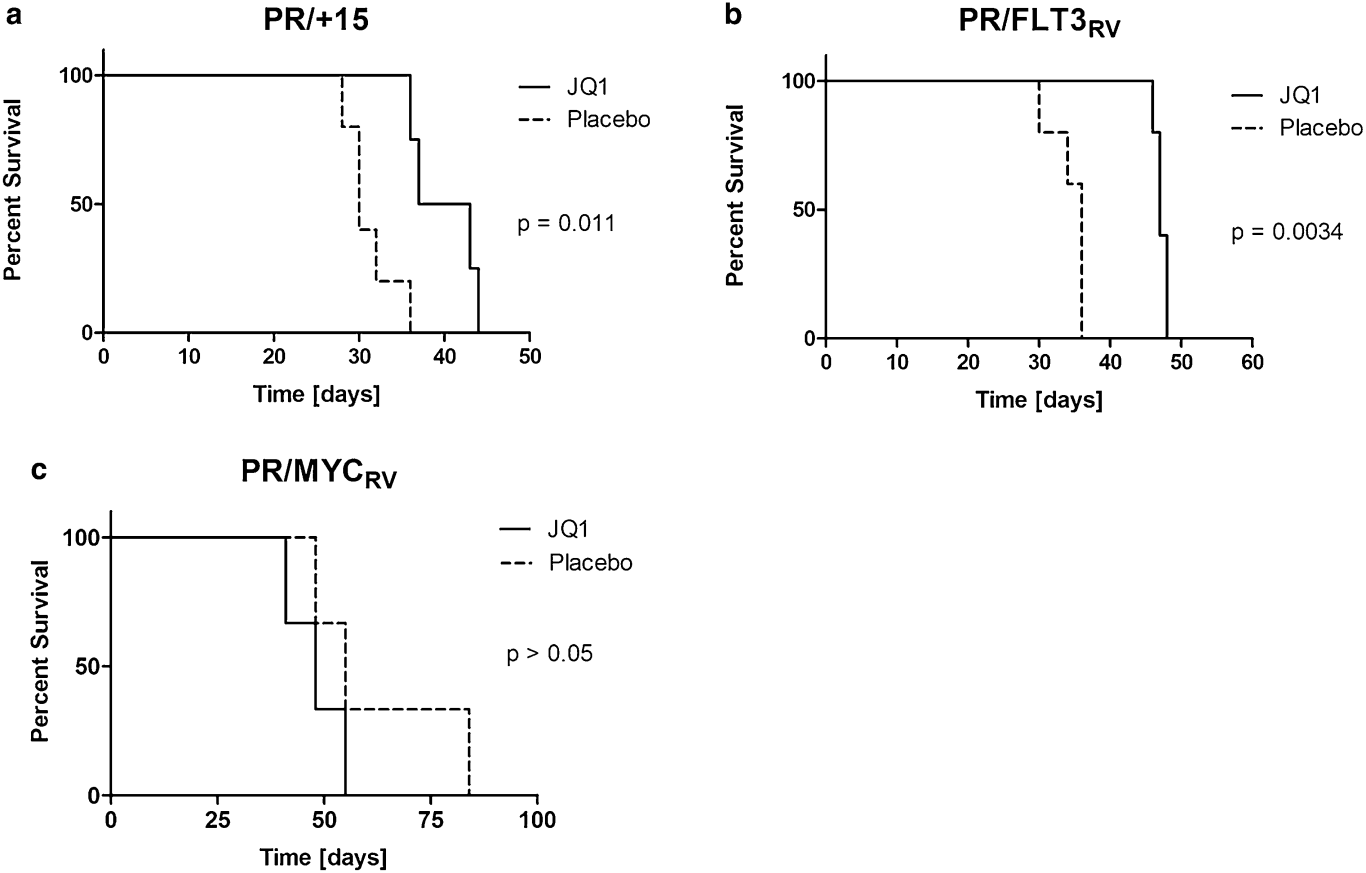
JQ1 prolongs survival in mice transplanted with *Myc*-overexpressing AMLs. **a** Kaplan–Meier curve comparing survival of FVB/n mice transplanted with PR/+15 leukemia treated for 21 days with JQ1 or placebo (*N* = 5 per group). **b** Kaplan–Meier curve comparing survival of FVB/n mice transplanted with PR/FLT3_RV_ leukemia treated for 21 days with JQ1 or placebo (*N* = 5 per group). **c** Kaplan–Meier curve comparing survival of FVB/n mice transplanted with PR/MYC_RV_ leukemia treated for 21 days with JQ1 or placebo (*N* = 3 per group)

Mice treated with JQ1 survived significantly longer than mice treated with placebo, whether injected with PR/+15 leukemia (Fig. 5a, *P* = 0.011) or PR/FLT3_RV_ leukemia (Fig. 5b, *P* = 0.0034). Of interest, in the group injected with PR/MYC_RV_, mice treated with JQ1 did not live longer than mice treated with placebo (Fig. 5c, *P* > 0.05). This result is expected since in the PR/MYC_RV_ leukemia, *MYC* expression is driven by a heterologous promoter, whereas JQ1 activity relies predominantly on downregulation at the endogenous *MYC* promoter. The PR/MYC_RV_^T58A^ leukemia did not engraft sufficiently for study in these JQ1 experiments.

## Discussion

MYC is commonly overexpressed in AML and has been shown to contribute to leukemogenesis [12, 14–16]. In this report, we compare specifically the effect of targeting MYC across AMLs that overexpress MYC through different mechanisms (trisomy of *MYC/Myc*-containing chromosomal regions, *FLT3*-*ITD* mutation, or gene amplification). Trisomy 8 and *FLT3*-*ITD* mutation have strong clinical relevance as they are the two most common mechanisms of MYC overexpression in human AML, and patients with *FLT3*-*ITD* AML have a particularly poor prognosis. We showed that shRNA knockdown of *Myc* in murine AMLs overexpressing MYC by trisomy 15 or *FLT3*-*ITD* prolongs survival and inhibits the ability of leukemic cells to predominate in vivo. We showed that VX-680, GDC-0941, and JQ1 significantly inhibit the growth of several human AML cell lines, while artemisinin does not. We further showed that the effect of VX-680, GDC-0941, and JQ1 on MYC levels varies across the AML cell lines tested, but we did not observe a discernible pattern of effect with respect to the mechanism underlying MYC overexpression. Thus, our experiments did not support our initial hypothesis that drug sensitivity would relate to the mechanism of MYC overexpression. JQ1 was observed to decrease MYC protein levels, and for several cell lines, the measured GI50 coincided with a sharp decrease in MYC protein levels. Additionally, JQ1 was generally more efficacious against AML cell lines that contained lower levels of MYC protein. Finally, we showed that JQ1 prolongs survival of mice injected with AMLs when *Myc* is overexpressed under control of its endogenous promoter, whereas GDC-0941 did not demonstrate efficacy against the MYC-overexpressing AMLs tested in our preclinical studies. Overall, our experiments demonstrated that targeting MYC in various MYC-overexpressing AMLs is an effective strategy that merits further development and that JQ1 appears to be a particularly promising drug in this regard.

In these experiments, we specifically compare drug efficacy across MYC-overexpressing AML cell lines stratified by mechanism of MYC overexpression. VX-680, which has been reported as synthetic lethal toward MYC [20], showed efficacy across all MYC-overexpressing cell lines except HL-60, which overexpresses MYC by gene amplification. There was no correlation between MYC levels and GI50s among the cell lines tested. In fact, HL-60 had the highest level of MYC protein of the cell lines tested and was the least sensitive to VX-680. Previous data suggest that higher MYC levels correlated with synthetic lethality with VX-680 [20], but this association was not observed in our experiments. This difference may have been due to the specific cell lines used, particularly with regard to effects of genes other than *MYC* that were not explored here. Assessing drug effects relative to doubling time, as we did herein, might also have contributed to our results in comparison with the prior study. Of note, we did observe decreased MYC level after VX-680 exposure in the MV4-11 and MOLM-14 cell lines, which both contain *FLT3*-*ITD* mutations. This is possibly due to the ability of VX-680 to inhibit the FLT3 expressed in these cells, which thereby may have contributed to a drop in MYC expression. Since clinical trials utilizing VX-680 have been terminated, testing of alternative aurora kinase inhibitors in MYC-overexpressing AMLs, particularly those with trisomy 8 or *FLT3*-*ITD*, may be of interest.

GDC-0941 showed efficacy against all cell lines, but this did not generally correlate with decrease in MYC levels. This lack of correlation suggests that growth inhibition in vitro by GDC-0941 does not reflect decreased MYC levels. Therefore, GDC-0941 does not appear to be a MYC-specific therapy in this setting. Furthermore, GDC-0941 was not efficacious in vivo against MYC-overexpressing leukemias. Given that GDC-0941 does not appear to be specific for MYC and did not demonstrate efficacy in our preclinical studies, these experiments do not support the continued exploration of this particular drug in the treatment of MYC-overexpressing AML. Of note, recent analysis suggests that overexpression of MYC may be a mechanism of resistance to PI3-kinase inhibitors [38].

In general, artemisinin showed minimal effect. GI50 was achieved for HL-60 and MV4-11. However, the relatively low GI50 against HL-60 may have been due to an early inflection point in the curve and may not reflect true efficacy. Nevertheless, the result may warrant further trials of artemisinin in the rare cases of AML with amplified MYC. In vivo testing was not undertaken due to limited drug effect in vitro. Overall, our experiments do not support the continued exploration of artemisinin as drug therapy against MYC-overexpressing AML.

JQ1 demonstrated strong efficacy across all cell lines. Among the drugs tested, JQ1 was the only compound effective at reducing MYC levels regardless of the mechanism of overexpression, and JQ1 doses near the GI50s were associated with a decrease in MYC levels. HL-60, the cell line with the highest basal levels of MYC, had the highest GI50, and we observed a positive correlation between GI50 and MYC level, suggesting that JQ1 may be particularly efficacious in AMLs with low to moderate MYC expression. Lower MYC levels may lead to increased susceptibility to growth inhibition by JQ1. In our preclinical in vivo studies, JQ1 demonstrated efficacy against leukemias with deregulated MYC, and this effect was lost when *MYC* was expressed under a heterologous promoter, indicating that downregulation at the *Myc* promoter is a major mechanism mediating JQ1 activity in these experiments. Together, these data support the role of JQ1 as an agent that lowers MYC protein levels and is thus able to inhibit growth of MYC-dependent leukemias, with an efficacy inversely correlated with MYC expression levels.

Previous work showed that JQ1 inhibits *MYC* transcription in a dose- and time-dependent manner, leading to the depletion of the c-MYC oncoprotein and selective downregulation of downstream transcriptional targets [26]. In addition, JQ1 may limit the activity of c-MYC by disrupting P-TEFb and transcription elongation [39, 40]. JQ1 has demonstrated antileukemic effects in vitro against a variety of human AML cell lines and primary patient samples [29, 41, 42], as well as in vivo against human AML xenografts [41] and murine MLL/AF9; Kras^G12D^ transplants [29]. These effects include growth inhibition [42], cell cycle arrest, and apoptosis [29], among others. Of additional interest, isocitrate dehydrogenase (IDH) gene mutations have been shown to initiate the development of AML by cooperating with oncogenic *FLT3* or *NRAS*. IDH mutant AMLs showed great sensitivity to JQ1, and western blots of IDH mutant leukemias treated with JQ1 showed a decrease in MYC levels [43]. JQ1 has also demonstrated efficacy against B-ALL cell lines with high-risk cytogenetics [44]. Another bromodomain inhibitor, I-BET, has shown efficacy against several MLL-fusion leukemias, including cell lines, murine leukemias, and primary patient samples [45].

Our data support the idea that targeting MYC may be beneficial for the treatment of most AMLs, regardless of mechanism of MYC overexpression, and that bromodomain inhibitors may be useful for this purpose until a direct MYC inhibitor can be developed. These experiments support future investigation of both direct and indirect MYC-targeting AML therapies and further establish *MYC* as an oncogene of central importance to AML pathogenesis and treatment.

## Electronic supplementary material

Supplementary material 1 (DOCX 273 kb)

Supplementary material 2 (DOCX 190 kb)

Supplementary material 3 (DOCX 843 kb)

Supplementary material 4 (DOCX 22 kb)

Supplementary material 5 (DOCX 22 kb)

Supplementary material 6 (DOCX 23 kb)
