# Supplementary material for: Direct and indirect targeting of MYC to treat acute myeloid leukemia
Source: Cancer Chemother Pharmacol. 2015 May 9;76(1):35–46. doi: 10.1007/s00280-015-2766-z (PMC4485702; doi:10.1007/s00280-015-2766-z)
Supplement: Supplementary file 1 — Supplementary material 1 (DOCX 273 kb) [file 280_2015_2766_MOESM1_ESM.docx]

**Supplementary Figure S1. Results of luminescence assay for compounds tested against seven human AML cell lines**


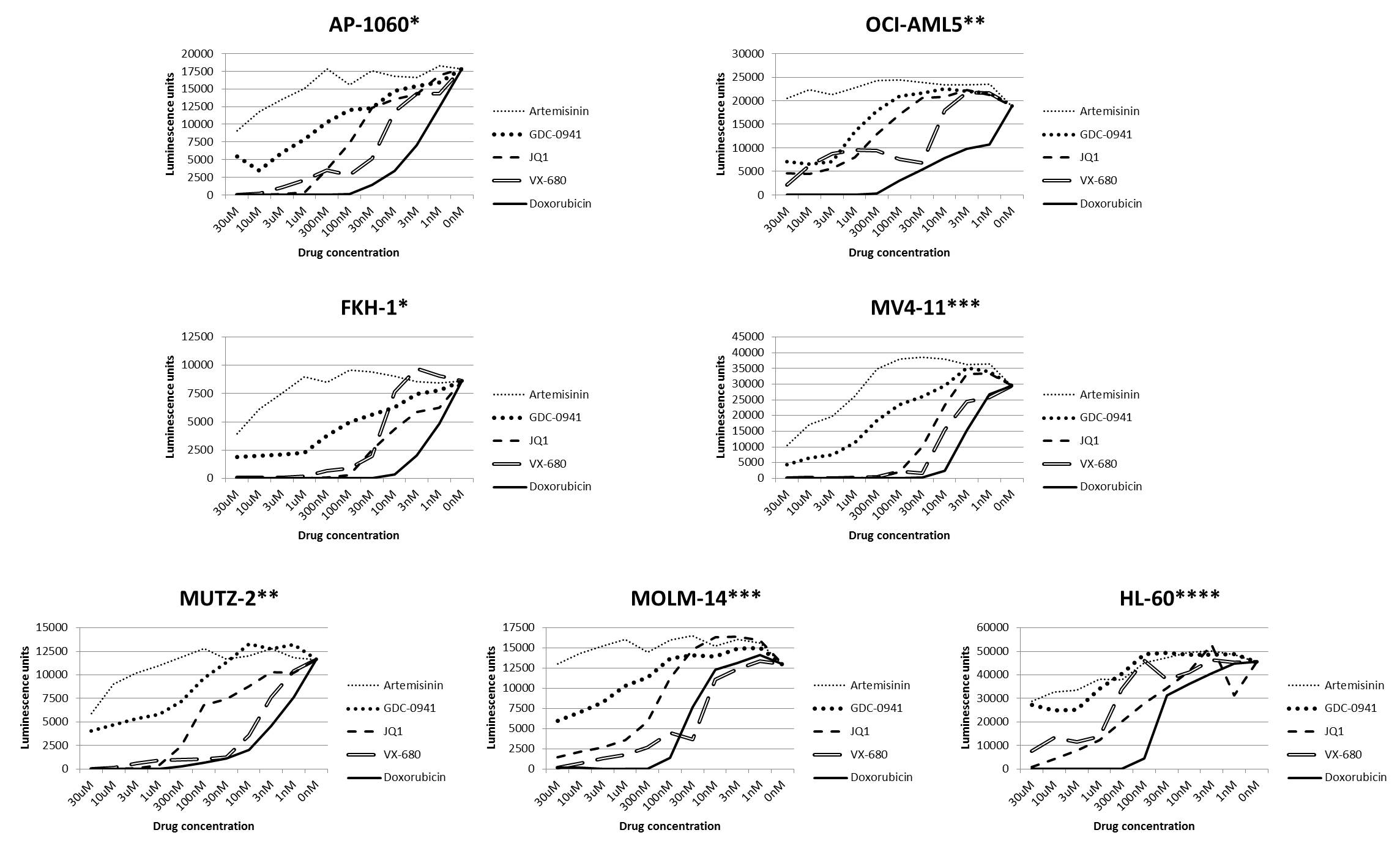


**Supplementary Figure S1: a**, AP-1060. **b**, OCI-AML5. **c**, FKH-1. **d**, MV4-11. **e**, MUTZ-2. **f**, MOLM-14. **g**, HL-60. Curves represent the average of two independent experiments. Luminescence at 0 nM drug concentration was determined by averaging five control wells. Luminescence units are reported as measured by a luminometer as part of the CellTiter-Glo assay. Cell lines are labeled by mechanism of MYC overexpression: *no known MYC overexpression; **trisomy 8; ***FLT3-ITD and gain of chromosome 8; ****MYC amplification.
