# Supplementary material for: Direct and indirect targeting of MYC to treat acute myeloid leukemia
Source: Cancer Chemother Pharmacol. 2015 May 9;76(1):35–46. doi: 10.1007/s00280-015-2766-z (PMC4485702; doi:10.1007/s00280-015-2766-z)
Supplement: Supplementary file 2 — Supplementary material 2 (DOCX 190 kb) [file 280_2015_2766_MOESM2_ESM.docx]

**Supplementary Figure S2: MYC protein expression, at basal levels and following exposure to the drugs**

**a**


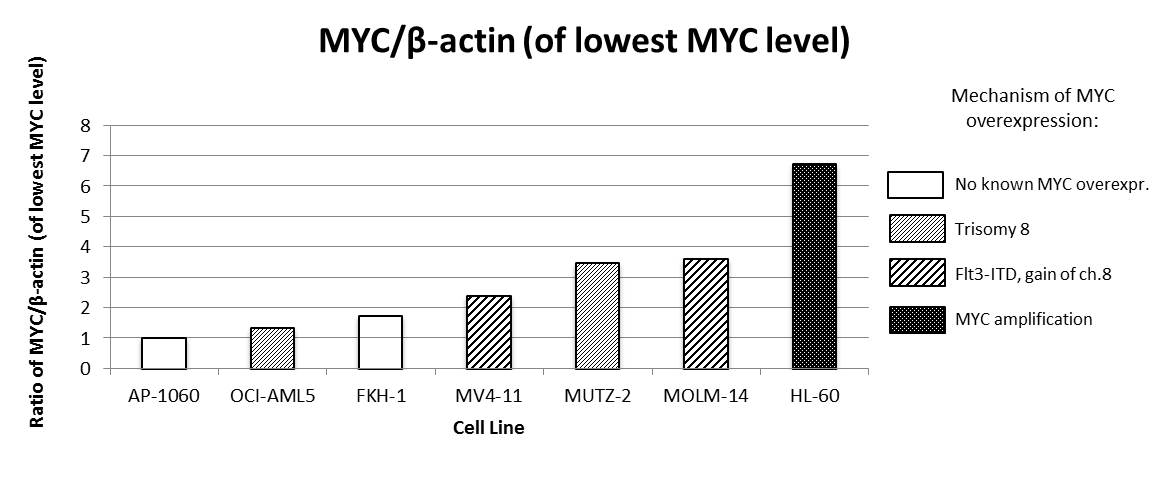


**
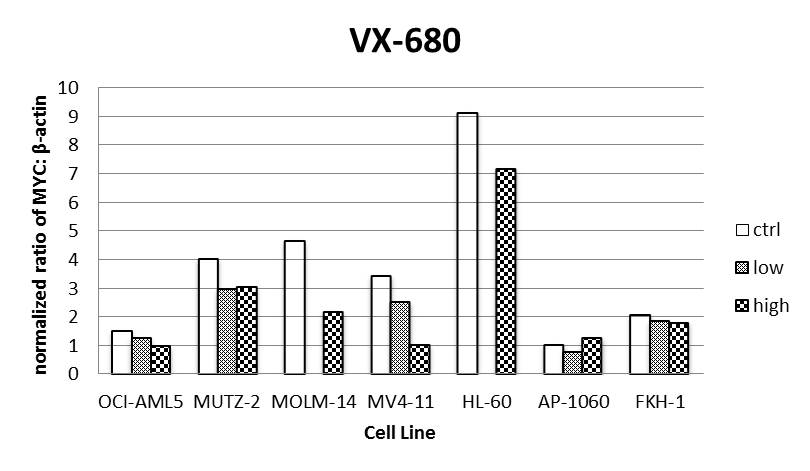
b**


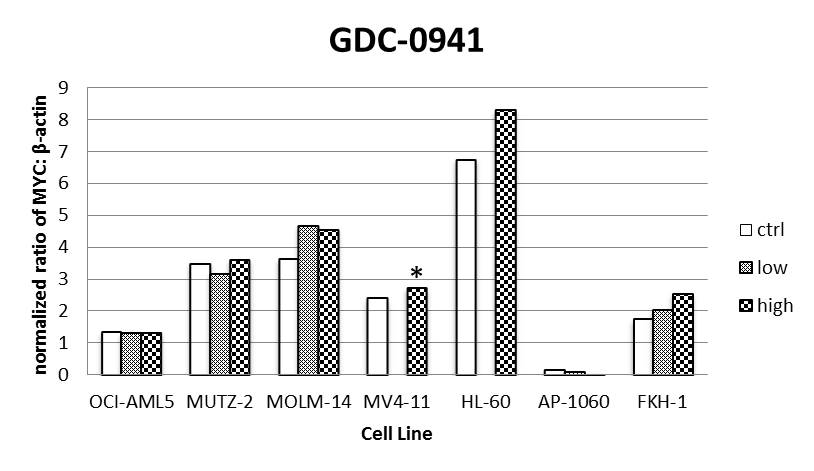
**c**


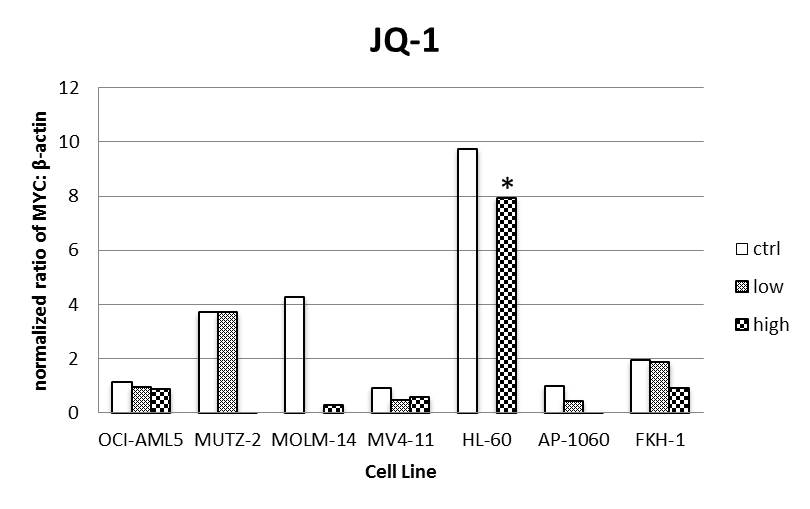


**d**

**Supplementary Figure S2:** **a**, MYC:β-actin ratios were determined from the Western blot presented in **Fig. 3a** (untreated samples were grown for one doubling time), and normalized to the cell line with the lowest MYC:β-actin ratio, AP-1060. **b**, MYC:β-actin ratios were calculated following treatment with VX-680 at concentrations spanning the GI50. **c**, MYC:β-actin ratios were calculated following treatment with GDC-0941 at concentrations spanning the GI50 (unless specified, see asterisk below). **d**, MYC:β-actin ratios were calculated following treatment with JQ1 at concentrations spanning the GI50 (unless specified, see asterisk below). Notes for **Figures S3b-d in electronic supplementary material**: in a few instances, only one drug concentration was investigated (above the GI50 in all cases); refer to **Fig. 3b** for the original Western Blots. *: in these two cases, the drug concentration tested isn’t immediately above the GI50 but rather another half-log unit higher, yet no decrease in MYC levels is observed at these higher drug concentrations (for MV4-11, GI50 for GDC-0941 was 212.7nM; for HL-60, GI50 for JQ-1 was 250.1nM).
