# Supplementary material for: Direct and indirect targeting of MYC to treat acute myeloid leukemia
Source: Cancer Chemother Pharmacol. 2015 May 9;76(1):35–46. doi: 10.1007/s00280-015-2766-z (PMC4485702; doi:10.1007/s00280-015-2766-z)
Supplement: Supplementary file 3 — Supplementary material 3 (DOCX 843 kb) [file 280_2015_2766_MOESM3_ESM.docx]

**Supplementary Figure S3. Luminescence results of the CellTiter-Glo assay, plotted over drug concentration, with best-fit logistic regression, associated GI50 values, and R^2^ correlation coefficients**


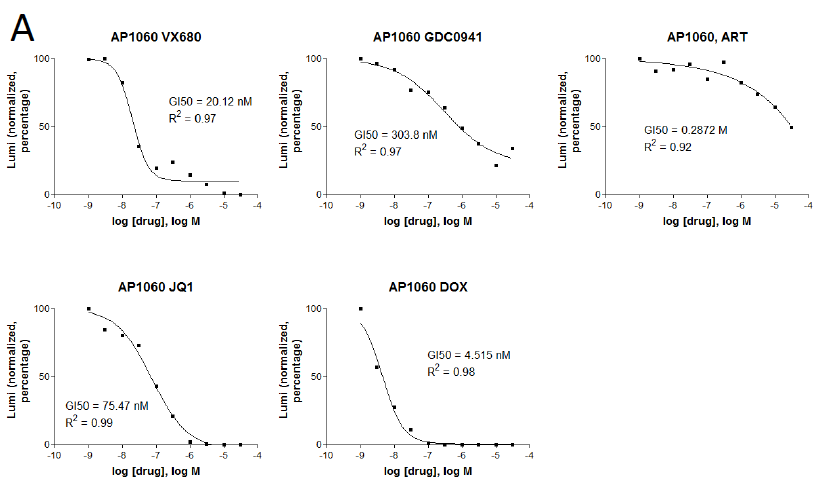


**a**


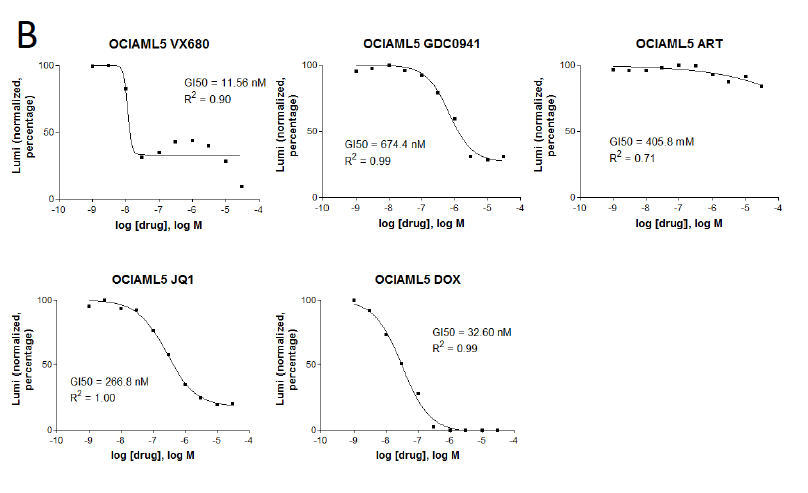


**b**


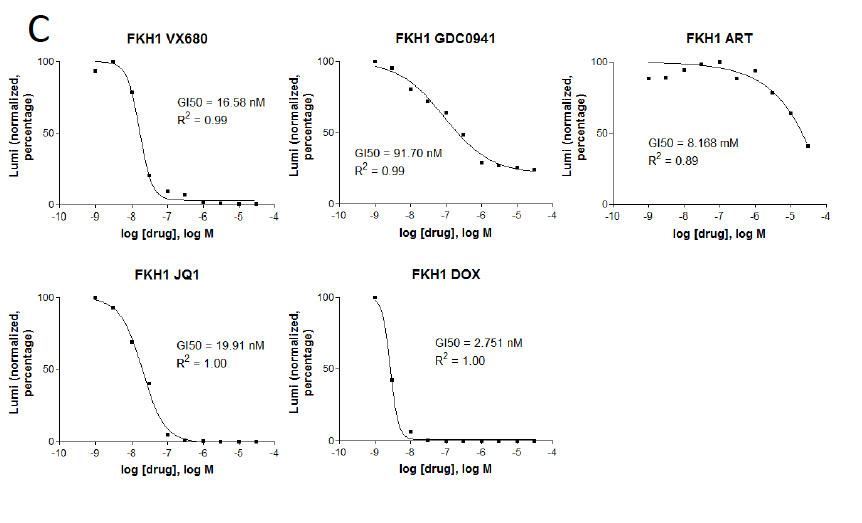


**c**


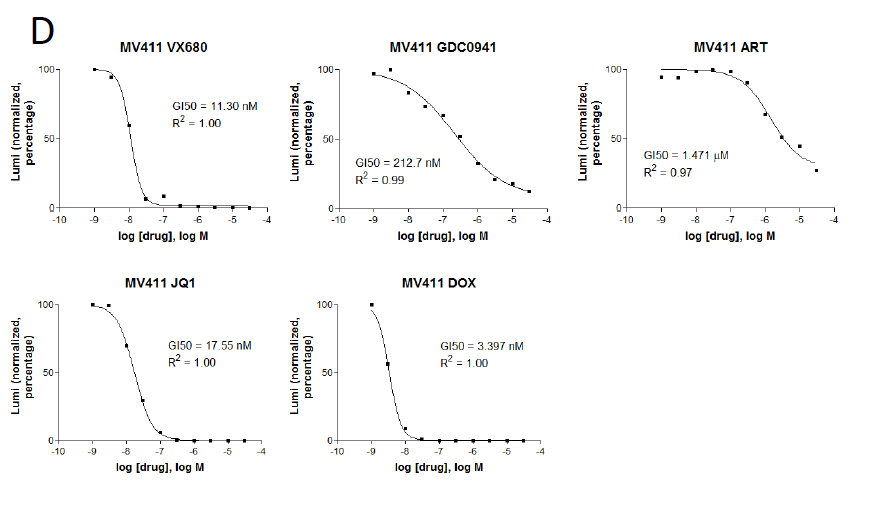


**d**


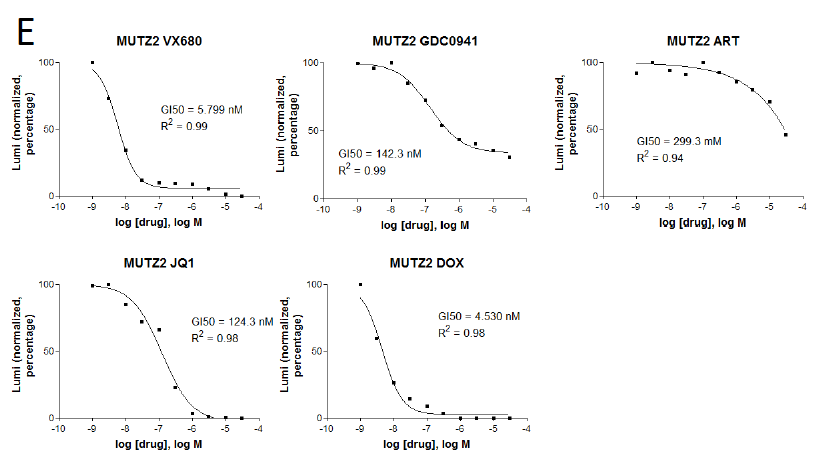


**e**


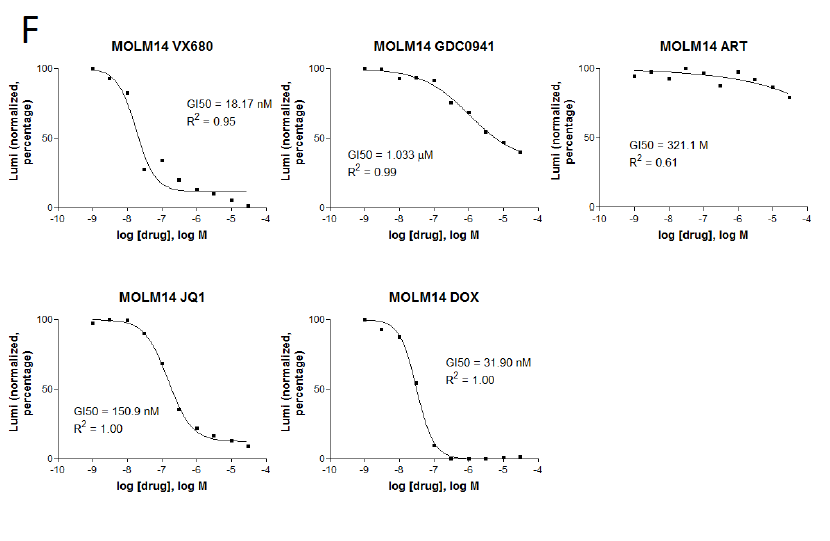


**f**


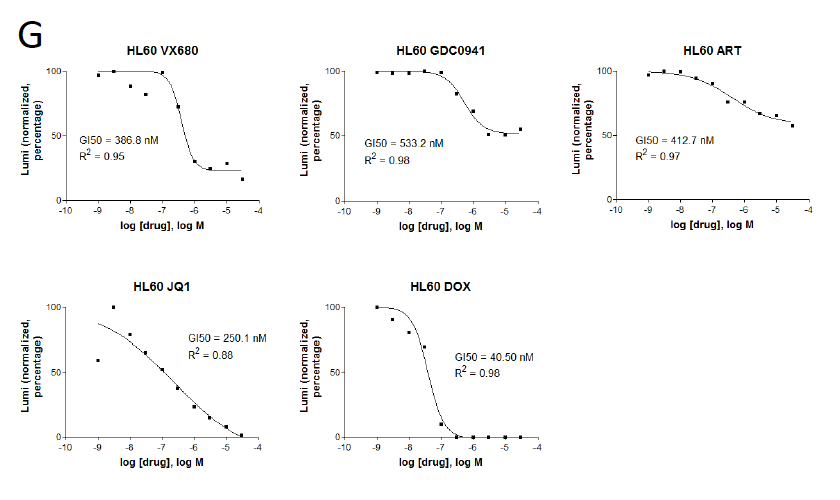


**g**

**Supplementary Figure S3: a**, AP-1060: no known MYC overexpression. **b**, OCI-AML5: characterized by trisomy 8. **c**, FKH-1: no known MYC overexpression. **d**, MV4-11: characterized by FLT3-ITD and trisomy 8. **e**, MUTZ-2: characterized by trisomy 8. **f**, MOLM-14: characterized by FLT3-ITD and trisomy 8. **g**, HL-60: characterized by MYC amplification. Abbreviations: ART, artemisinin; DOX, doxorubicin.
