# Supplementary material for: Direct and indirect targeting of MYC to treat acute myeloid leukemia
Source: Cancer Chemother Pharmacol. 2015 May 9;76(1):35–46. doi: 10.1007/s00280-015-2766-z (PMC4485702; doi:10.1007/s00280-015-2766-z)
Supplement: Supplementary file 4 — Supplementary material 4 (DOCX 22 kb) [file 280_2015_2766_MOESM4_ESM.docx]

**Supplementary Table S1.** **Measured GI50 concentrations for compounds tested against human AML cell lines.**

| **Cell Line** | **VX-680** | **GDC-0941** | **Artemisinin** | **JQ1** | **Doxorubicin** |
| --- | --- | --- | --- | --- | --- |
| **AP-1060*** | 20 nM | 300 nM | *NR* | 75 nM | 4.5 nM |
| **OCI-AML5**** | 12 nM | 670 nM | *NR* | 270 nM | 33 nM |
| **FKH-1*** | 17 nM | 92 nM | *NR* | 20 nM | 2.8 nM |
| **MV4-11***** | 11 nM | 210 nM | 1.5 µM | 18 nM | 3.4 nM |
| **MUTZ-2**** | 5.8 nM | 140 nM | *NR* | 120 nM | 4.5 nM |
| **MOLM-14***** | 18 nM | 1.0 µM | *NR* | 150 nM | 32 nM |
| **HL-60****** | 390 nM | 530 nM | 410 nM | 250 nM | 41 nM |

**Supplementary Table S1:** Values are averaged from two independent experiments and rounded to two significant figures. Values “not reached” were extrapolated to be higher than 30 μM, the highest tested drug concentration, and thus were not reported. *NR*, not reached. Cell lines are labeled by mechanism of MYC overexpression: *no known MYC overexpression; **trisomy 8; ***FLT3-ITD and gain of chromosome 8; ****MYC amplification.
