# Supplementary material for: Direct and indirect targeting of MYC to treat acute myeloid leukemia
Source: Cancer Chemother Pharmacol. 2015 May 9;76(1):35–46. doi: 10.1007/s00280-015-2766-z (PMC4485702; doi:10.1007/s00280-015-2766-z)
Supplement: Supplementary file 5 — Supplementary material 5 (DOCX 22 kb) [file 280_2015_2766_MOESM5_ESM.docx]

**Supplementary Table S2.** **Rank-order of sensitivity, most to least sensitive, measured by GI50 value, of seven AML cell lines to the tested drugs.**

| **Rank** | **VX-680** | **GDC-0941** | **Artemisinin** | **JQ1** | **Doxorubicin** |
| --- | --- | --- | --- | --- | --- |
| **1** | **MUTZ-2**** | **FKH-1*** | **HL-60****** | **MV4-11***** | **FKH-1*** |
| **2** | **MV4-11***** | **MUTZ-2**** | **MV4-11***** | **FKH-1*** | **MV4-11***** |
| **3** | **OCI-AML5**** | **MV4-11***** | - | **AP-1060*** | **AP-1060*** |
| **4** | **FKH-1*** | **AP-1060*** | - | **MUTZ-2**** | **MUTZ-2**** |
| **5** | **MOLM-14***** | **HL-60****** | - | **MOLM-14***** | **MOLM-14***** |
| **6** | **AP-1060*** | **OCI-AML5**** | - | **HL-60****** | **OCI-AML5**** |
| **7** | **HL-60****** | **MOLM-14***** | - | **OCI-AML5**** | **HL-60****** |

**Supplementary Table S2:** A rank of 1 represents the most sensitive cell line, and a rank of 7 represents the least sensitive cell line. Dashes represent cell lines with GI50 values higher than the drug concentrations tested in these experiments. Cell lines are labeled by mechanism of MYC overexpression: *no known MYC overexpression; **trisomy 8; ***FLT3-ITD and gain of chromosome 8; ****MYC amplification.
