# Supplementary material for: Direct and indirect targeting of MYC to treat acute myeloid leukemia
Source: Cancer Chemother Pharmacol. 2015 May 9;76(1):35–46. doi: 10.1007/s00280-015-2766-z (PMC4485702; doi:10.1007/s00280-015-2766-z)
Supplement: Supplementary file 6 — Supplementary material 6 (DOCX 23 kb) [file 280_2015_2766_MOESM6_ESM.docx]

**Supplementary Materials and Methods**

*Constructs and bacterial subcloning*

Bacterial plasmid constructs containing shRNA sequences were obtained from the laboratory of Dr. Scott W. Lowe at Cold Spring Harbor: pMSCV-miR30.1224-SV40-mCherry (MLC) vector containing the anti-Renilla.713 sequence, pMSCV-miR30.1224-SV40-GFP (MLS) vector containing the anti-RPA3.457 shRNA sequence, the anti-Myc.1888 sequence, or the anti-Myc.1891 sequence, and pMSCV-miR30-Puromycin-IRES-GFP (LMP) vector containing the anti-Myc.2105 sequence. These plasmids were transformed into DH5α. Individual colonies were mini-prepped (Bioneer cat#K-3030) and sequenced. After validation, MaxiPreps were prepared (Qiagen cat#12663) for subcloning. To isolate the shRNA fragments, 20 μg of plasmid was digested with XhoI and EcoRI, and gel purified. To isolate the mCherry-containing backbone, 5 μg of MLC-Ren.713 plasmid was digested with XhoI and EcoRI, and gel purified. Ligation was performed at 15°C overnight using the T4 DNA ligase in a 1:4 backbone:insert ratio.

*Western Blot Calculations*

Chemiluminescent Substrate by Supersignal West Pico and the Bio-Rad Chemidoc XRSplus machine were used for imaging the blots, and the Imagelab 3. 0 software from Bio-Rad used for quantifying bands intensities. MYC:β-actin ratio was calculated for each cell line. Values were normalized to the MYC:β-actin ratio of AP-1060, the cell line with the lowest MYC:β-actin ratio (**Figure S3a in electronic supplementary material**). For each cell line, the MYC:β-actin ratio from the control blot (**Fig. 3a**) was divided by the MYC:β-actin ratio of the control lane from each cell line blot (**Fig. 3b**). The result was referred to as the multiplication factor for each cell line. For each blot, the MYC:β-actin ratio for the control, low and high drug concentration lanes was multiplied by each cell line’s multiplication factor. The resulting value represented the normalized MYC:β-actin ratio. The normalized MYC:β-actin ratio was divided by the AP-1060 MYC:β-actin ratio. The MYC:β-actin ratios, normalized to the AP-1060 control ratio, were plotted on a graph for each drug (**Figures S3b-d in electronic supplementary material**). For each drug, the GI50 of each cell line was plotted against the normalized MYC:β-actin ratio to assess the relative sensitivity of each drug according to MYC level (**Fig. 4**).
